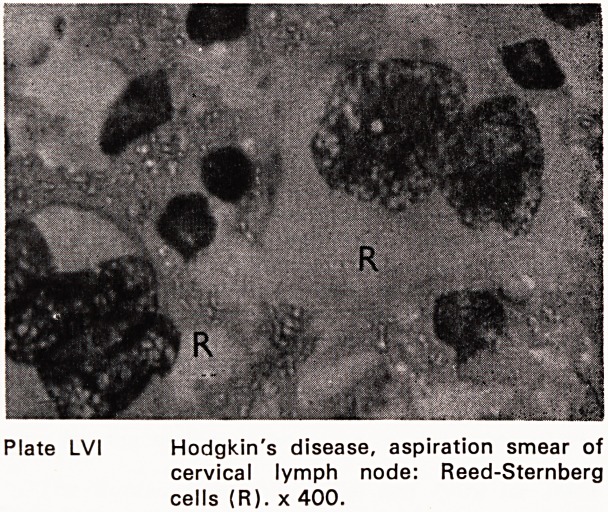# 'Through a Glass Darkly' (The Development of Needle Aspiration Biopsy)

**Published:** 1974-10

**Authors:** A. J. Webb

**Affiliations:** Consultant Surgeon, United Bristol Hospitals


### Through a glass darkly*

### (The Development of Needle Aspiration Biopsy)

A. J. Webb, M.B., Ch.M., F.R.C.S., M.I.A.C.
Consultant Surgeon, United Bristol Hospitals

Mr. Dean, Ladies and Gentlemen, "I was never more surprised, and I think I can honestly add, never more gratified than when I heard that I had been chosen to deliver the Long Fox Lecture. It is true that a contemplation of the names of my predecessors and the lectures they have delivered has filled me with a sense of my own unfitness for so weighty a responsibility". Although these are not my words, I echo their sentiments. They comprise the opening sentences of the 14th lecture delivered by Dr. Carey Coombs, Physician to the Bristol General Hospital; an eminent cardiologist. If he felt overawed you can imagine how I feel!

My terms of reference this evening, for the 62nd Lecture, derive from a meeting held in the Chapter House of Bristol Cathedral on 25th May, 1902. Long Fox had died on 25th March of that year. His friends, and by all accounts he possessed 'troops of them', met and resolved that annually a lecture should be given in his memory. To quote Harrison (1906):

> "the subject of which should be of a very marked professional bearing and of more or less utility to the hard working members of the medical profession".

Perhaps then, for a brief moment, we should consider our hero, Edward Long Fox Jun., Born 1832, Died 1902, in order to set the scene: who was he? why do we honour him?

According to Munro Smith (1917), he was of middle height, strongly made, energetic and with a quick determined walk. Neat of dress, with fresh bright eye, ruddy complexion, set against his black hair and whiskers. Sir Henry Newbold found him "a great doctor with a fine intelligent face". Reputedly a good host, he told anecdotes in a forceful, clever way. I feel sure that the younger members of this audience will have quickly detected a typical surgeon! —not so—from 1857 until 1877 he graced the Royal Infirmary as Physician.

From personal tributes this picture is amply confirmed. Arthur Rendle Short who gave this lecture on 29th November, 1929, when I lay swimming in my mother's womb, met Long Fox but once. All the medical freshmen of his day were invited to Church House, Clifton Hill, for coffee and cakes. Long Fox expressed the hope that not only would they be good students, but adjured them to remember that "character matters more than learning". He loved gatherings of his professional brethren and in the view of Nixon (1930), was one of the most scientific doctors of his day.

Long Fox, by any standards, was a 'winner'.

**The 62nd Long Fox Memorial Lecture, delivered in the University of Bristol on 15th November, 1973.*

I have chosen to address you on the origins and development of clinical cytology. Although none of the previous lecturers, even those who considered malignant disease, have mentioned the subject, its basis, *microscopy,* can be associated with Long Fox. He was a colleague and admirer of William Budd. Budd reported on early microscopy (Budd, 1841-2) and his prophetic views on the infective origins of typhoid, cholera, tuberculosis and scarlatina antedated Pasteur and Koch by several years. Long Fox shared Budd's interest in the so-called 'animalcules'.

Firstly, I must define cytology, or the study of cells; it derives from the Greek 'Kytos'—a hollow urn, jar or vessel—used in the sense of a cell (Stedman's Dictionary, 1972). Clinical cytology is the application of this study within clinical practice. There are two styles: exfoliative and non-exfoliative cytology, the latter being:

> "the examination of cells obtained by needle or drill biopsy in solid organs or tissue masses or from smears made from the cut surface of such material freshly removed by surgical operation".
>
> (Bamforth, 1966)

Exfoliative cytology is the scrutiny of spontaneously cast off cells as in sputum or urine. The most popular aspect of cytology is the cervical smear which involves scraping the cervix uteri—and is sensibly regarded as a combination of both types. Tissue cytology is the 'cinderella', being far less popular than the exfoliative style.

To introduce the subject may I quote two case histories, both of which demonstrate how tissue cytology can be advantageous. *Case 1:* Mrs. P.B., aged 23, attended her doctor with a small lump associated with the left breast in February 1971, but failed to complete arrangements to attend my clinic at the Royal Infirmary because—she stated—of impending divorce proceedings. By September 1971, she was so dyspnoeic that I was asked to see her at home. The lump which had increased in size was above and deep to the breast. Clinically it lay in the chest wall and there was a total left pleural effusion. Fine needle aspiration biopsy performed in the bedroom provided diagnostic material. An hour later, I was in possession of the probable diagnosis, having stained and viewed the slides in my small domestic laboratory. This was no breast cancer or lymphoma but a Ewing's tumour. I am aware of the controversy surrounding this pathological entity, but that is what it was (Price, 1973). The subsequent biopsy led to a histological appearance of closely packed cells. The needle aspiration had provided immediate accurate information upon which to base future management.

*Case 2:* Mrs. P.H., aged 62, presented at my clinic with a three-year history of a left parotid swelling. Fine needle biopsy in the clinic provided the answer. (Plate XXXVII) At operation, against all expectations,

Plate XXXVII Case 2; aspiration smears, showing amorphous debris, lymphocytes and oncocytes (C). x 100 Giemsa.

the tumour was found to lie deep to the facial nerve divisions and looked far from benign. But, armed with the cytological diagnosis, we were fortified in our attempts to extract the tumour from between the branches of the nerve. In this case, the preliminary cytological diagnosis ensured the correct surgical procedure.

Plate XXVIII Case 2; histology of the tumour. The characteristic appearance of papillary cystadenoma lymphomatosum. H & E x 200.

The histology (Plate XXXVIII) confirmed the cytology—a papillary cystadenoma lymphomatosa—adenolymphoma or Warthin's tumour, although the latter has little claim to the eponym (Nicholson, 1922; Warthin, 1929).

These two cases exemplify diagnosis from cells, obtained in a simple manner from a clinical lesion—in other words, a form of biopsy, which is, by definition:

> "The removal of any tissue (or cells) from a living subject for diagnostic examination".
> (McGraw & Hartmann, 1933)

In contrast to a section made from fixed tissue—histology—the classical and widely accepted mode of examining biopsy material (Bloodgood, 1931), in these quoted cases a smear of cells was also studied. There are three basic techniques employed in tissue cytology, to which I shall refer during this lecture.

1. The tissue imprint (Plate XXXIX), where material is dabbed on to a slide leaving a representative layer of cells.

Plate XXXIX Technique of making a tissue imprint.

2. The scrape-smear, which is very similar, except that cells are scraped from the cut surface and a smear is fashioned.
3. Fine needle aspiration biopsy (Plate XL) using a 20 ml disposable syringe and 23 gauge needle and constant suction. Cells are drawn into the needle bore and later blown onto the slide and spread out.

Plate XL Fine needle aspiration incorporating constant suction: case of recurrent cystosarcoma phyllodes.

Fine needle biopsy is the most useful, and also the most controversial. It is defined as:

> "the withdrawal of cells or bits of tissue through a needle by means of a negative pressure"
> (Godwin, 1955-56)

Whatever the technique used, the cells are spread on to a slide, and in my practice, *rapidly air-dried.* Fixation in methanol is followed by a Romanowsky stain—usually Giemsa's. The whole procedure from aspiration to microscopy need take only twenty minutes—sometimes less.

How has this discipline developed and, most important, how accurate can it be? Obviously, before cells could be studied some means of seeing them had to be found. Hence my story must continue with a few highlights in the establishment of microscopy and the Cell. Theory. Time allows only the briefest consideration

**Microscopy**

It is well substantiated that, around 1590, in Middelburg, Zealand, Holland, Hans and Zacharias Jannsen devised the compound microscope, combining two convex lenses (Bradbury, 1967). Galileo quickly adopted the principle for his telescope but he knew also that by lengthening the distance between the lenses this arragement functioned as a miscroscope. The first truly scientific microscopist was Robert Hooke, whose Micrographia of 1665 is a classic. An ugly genius, by all accounts, he befriended Samuel Pepys who wrote on 20th January, 1665:

"To my bookseller's and there took home Hooke's Book of Microscopy, a most excellent piece, of which I am very proud".

Hooke, in Observation XVIII, concerning sections of cork, wrote:

"these pores or cells were not very deep but consisted of a great many boxes".

Also, concerning the plant stem:

"For in several of these vegetables, whilst green, I have with my microscope plainly enough discovered these cells or pores filled with juices".

Hooke discovered and named the vegetable cell and even measured their size with his primitive micrometer.

His place in microscopy has previously been the subject of a superb lecture to this Medical School by Dr. Jeffery Boss and I commend his paper to all (Boss, 1965).

The famous microscopists of the mid 17th century were Nehemiah Grew, Hooke's successor in the Royal Society, Borellus, Malpighi, Swammerdam of Leiden, and last but not least, the "great amateur", Antoni van Leeuwenhoek (Plate XLI). Van Leeuwenhoek used a simple microscope and ground his own lenses from quartz. As Van der Star (1953) has demonstrated, they were unbelievably good. Born in Delft (1632), he lived until 1723—in itself an achievement —and was a linen draper; in contemporary terms he was uneducated, but in 1673 Renier de Graaf (of ovarian follicle fame) wrote to the Secretary of the Royal Society of London in the following vein:

"A certain most ingenious person here named Leeuwenhoek has devised microscopes which far surpass those which we have hitherto seen manufactured by Divini and others".

(Divini was a famed contemporary Italian insturment maker.)

Plate XLI — Delft; a commemorative plaque to Antoni van Leeuwenhoek.

The rest is history; Antoni was elected F.R.S. in 1680 and undoubtedly saw bacteria and protozoa (Letter to Royal Society, 9th October, 1676): his "very little animalcules".

Compound microscopes possessed, at high power, several optical defects, in the nature of chromatic and spherical aberration, and many mistaken observations were made: microscopy in the 18th century fell into disrepute—a "true dark age". Around 1790, Francois Beeldsnyder (1775—1808), a colonel of cavalry, constructed achromatic lenses (Bradbury, 1967). Sadly, at high power their resolving power was poor and diffraction haloes rendered any object globular or fibrillar in appearance. There ensued a short era of microscopy known as 'globulism'. The globulists described artefacts and the first illustration of tumour microscopy (Home, 1830) was a 'globulistic triumph of error'.

Happily, all was corrected by the sound theoretical and practical optics of Joseph Jackson Lister (1826), who designed a system free from spherical and chromatic abberation. His paper, with Hodgkin (1827), and his own to the Royal Society (1830) initiated modern microscopy. The British, as so often, were sceptical, but not so the Germans, Austrians and French, who adopted his principles and made excellent cheap microscopes. From 1830 onwards the renaissance of microscopy flowered and these countries pursued microscopy intensely.

Until this time, surgeons made their diagnoses of tumours by clinical examination or device and by macroscopy of the tissue. Early English surgeons, Hey, Abernethy and Wardrop classified the macroscopy of tumours. Scirrhous referred to hard or fibrous lesions —whereas soft or mushy cancer, encephaloid or brain-like tissue was termed 'fungus haematodes'. Not surprisingly, Abernethy (1817) wrote of uterine fibroids:

> "they have something of the structure of cancer and yet are not cancerous".

Concerning breast lumps, Sir Astley P. Cooper, Surgeon to four monarchs, illustrated macroscopy; his impression of fibro-adenoma and cystic mastopathy in present terminology are beautifully clear. In his day, mastectomy was often performed for benign breast lesions, thought on clinical grounds to be cancerous (Cooper, 1829).

Associated with the renaissance of microscopy was the formulation of the Cell Theory—in all probability heralded by Morgagni with his famous book of 1761 'De Sedibus et Causis Morborum'. He correlated clinical features with post mortem findings and proposed that disease processes occurred in organs. Morgagni's precision, coupled with the brief appearance of Xavier Bichat, who by dissection, found 21 tissues in the body, led to the cell theory (Bichat, 1801). There is much written on the origins of this landmark and some observers have failed to receive the credit due to them. For instance, Purkinje of Breslau, by simple and compound microscopy, recognised plant and animal cells and defined protoplasm (Hughes, 1954). His pupils, Henle, Schwann and Valentin became famous, and Purkinje also persuaded Johannes Müller to pursue human microscopy.

The formal statement of the Cell Theory by Schleiden (1847) and Schwann (1847) is to any student of their times, somewhat of an anticlimax since neither were men of great stature. Schwann studied microscopy for a mere five years and succeeded in propagating two false doctrines.

1. He named the nucleus—'cytoblast'—and asserted that a daughter cell could arise within the parent cell.
2. He outlined free cell formation within a vital amorphous tissue substance termed 'Cytoblastema', derived from blood vessels.

For some years, many well known observers, including Virchow, Von Kolliker, Bennett and Henle subscribed to these misconceptions. Yet the most important advance derived from the Cell Theory was (Cameron, 1952):

> "The resolution of the complexity of morbid processes into simplified versions referrable to DISORDERS OF CELL LIFE".

In short, Cellular Pathology (Virchow, 1860), a concept which is forever associated with Rudolph Virchow. It is of interest and no little importance that Robert Remak, an ill-fated, ill-used Jew, outlined the Cell Theory—rejected cytoblastema and enunciated the correct origin of carcinoma from epithelium many years before his illustrious contemporaries (Kisch, 1954). His name is today only occasionally remembered in connection with non-medullated nerve fibres. He deserved better than this.

Around 1835-1845, microscopy was introduced into medical practice; in Germany, Johannes Müller (1838) published the first valid illustrations of tumour cells. John Hughes Bennett brought the microscope to British medicine in 1841, after study in Vienna with Gruby, and in Paris with Donné. Gruby later settled in Paris and founded mycology; Donné recognised cells in colostrum, squames in the vaginal smear and described Trichomonas vaginalis.

Bennett extended the microscopy of tumours, and wrote, somewhat prophetically:

> "The whole subject of tumour microscopy has yet to be worked out and it is desirable that some young surgeon would dedicate his time and energies to the task".

His book, "On Cancer and Cancroid Growths", appeared in 1849 and from it we see that he was both a skilled histologist and cytologist. Both techniques were used: Bennett was one of the few microscopists of this era to provide technical details on how to make cytological smears. Purely on technical grounds, the microscopy of smears was difficult; the cells were unfixed and unstained. Often 1% acetic acid was used to accentuate cellular features.

Lionel Smith Beale (1828-1906), a Londoner, was a supreme 19th century microscopist; some of his illustrations are superb and have been admired by countless modern cytologists. However, towards the 1870's, histology supervened and cytology was rejected; sections became more reliable due to microtomes and the introduction of stains invented by Ehrlich and others (Conn, 1925). Romanowsky stains for smears were not available until the early 20th century, hence the total eclipse of cytology.

**Needle biopsy**

What of the origins of needle biopsy? Skey (1850) advocated puncture of a doubtful breast lump in case it turned out to be a cyst, but discounted microscopy. Paget (1853) and Erichsen (1853) alone were in favour of aspiration biopsy and microscopy. Erichsen from University College Hospital proclaimed that microscopy was the only guide to the nature of a tumour and reported seven examples of mastectomy performed for chronic abscess simulating scirrhous cancer.

Paget (1853) has not been appreciated for his skill as a cytologist: to quote from his 'Lectures on Tumours':

> "Many of the cells of cancers, for example, may be somewhat like gland cells or like epithelial-cells, yet a practised eye can distinguish them even singly and much more plainly their grouping distinguishes them; they are heaped together disorderly and seldom have any lobular or laminar arrangements such as exists in the natural glands or epithelia".

Papanicolaou said it no more clearly 100 years later.

Augustin Prichard (1863), a B.R.I. Surgeon, pursued microscopy and used the grooved needle to assess breast lumps. In his fascinating little book "Ten years of operative surgery in the provinces—875 operations", he provided a clear description of the cytology of fat necrosis.

The earliest report of needle biopsy is probably by Kun (1847), a physiologist from Strasbourg, his description reads:

> An exploring needle, having at its extremity a small depression with cutting edges. On plunging this into the tumour one can extract a minute portion of tissue—in this manner a microscopic examination can be practised".

The next significant reference to needle aspiration concerned lymph nodes; a report from Captain Greig and Lieutenant Gray in 1904. They had observed motile trypanosomes in smears from biopsied nodes. They extended this examination to fluid aspirated from nodes. Over the next twenty years, node aspiration was recognised as a valuable means of demonstrating filariasis, bubonic plague, and spirochaetes in secondary syphilis. The systematic diagnosis of lymph node pathology by aspiration cytology came from Guthrie of Johns Hopkins Hospital in 1921—using air dried films and Romanowsky staining. Later South American and European workers adopted the method, especially for Hodgkin's Disease.

Thereafter its development was progressive but sporadic. During the later 1940's and early 1950's, aspiration biopsy of lymph nodes, salivary tumours, breast lumps, goitres and skeletal lesions became established in Europe, but, as Söderström (1966) remarked:

> "It has not gained general acceptance—the attitude to the method has been said to vary between over-enthusiasm and absolute rejection—in many centres it is virtually unknown".

In the words of Von Haam (1962), it remains a "frontier field of cytology". A few American centres, notably the Memorial Hospital, New York, began aspiration biopsy in the late 1920's at the instigation of James Ewing. By 1956, they were performing annually 2,500 biopsies from all services. Their technique is regarded by some as over-elaborate (Cardozo, 1971) and they endeavoured to obtain material for histology and cytology; the staining method is haematoxylin and eosin.

There was barely any interest in Great Britain.

What of imprints and scrape smears? Imprints were invented in the 1880's by Ehrlich and Lowit, to be rediscovered in the 1930's. Perhaps here, England may take some credit. In the 1920's and '30's, Professor Leslie Dudgeon with his associates at St. Thomas's Hospital—one (Mr. N. R. Barrett) happily still alive—produced classic work on tissue scrape smears (Dudgeon and Patrick, 1927; Dudgeon and Barrett, 1934) fixed wet with Schaudinn's fluid. Perhaps his death in 1939 prevented an earlier propagation of cytology, for even in his day, the very bases of cytological diagnosis were unacceptable to many—an attitude summarised by Sir John Bland-Sutton (1922):

> "in the appearance of a cell from cancer—there is nothing characteristic of the disease, nothing that would lead a pathologist to identify it as a malignant cell".

Plate XLII Dr. Paul Lopes Cardozo of Leiden; a leading international authority on tissue cytology.

Nevertheless, in Europe (Plate XLII) and parts of America, imprints gained an important though scattered support. In France, Guy and Colette Castelain commencing in 1940 on a whole range of pathological material, reported on 10,000 tumours by 1971, and have in a series of articles in 'La Presse Medicale' published many beautiful cytological illustrations of air-dried Giemsa-stained smears (Castelain and Castelain, 1957).

For Bristol, there is an important association with tumour imprints. Dr. J. N. P. Davies delivered this lecture in 1962 and hinted at the African or Burkitt Lymphoma. The pathologist who contributed greatly to its elucidation is Denis Wright, a Bristol graduate. Denis Wright used imprints for morphology and cytochemistry (Wright, 1963). Several reports accord imprints a 95% accuracy rate for fresh biopsies. Especially for lymph nodes, imprint cytology complements the histology in a remarkable way (Mavec, 1967).

Without doubt, the true modern renaissance of clinical cytology is attributable to Babes and Papanicolaou. Largely due to Papanicolaou, exfoliative cytology of the female genito-urinary tract, sputum, urine and effusions is now a world-wide discipline. It is significant that Papanicolaou's studies during the 1940's, first found support among the clinicians, not pathologists; this is fully understandable. His stain, which is complicated, took two years to develop; the fixation is wet, hence the cells are shrunken compared with Giemsa stained preparations. In the latter, the smear being air-dried, the cells are well spread. The chromatin form is also quite different.

**Personal experience**

To conclude, may I briefly present some of my own studies over the past ten years with some examples of how I have found cytology useful in clinical practice.

**Salivary gland lesions**

Considering salivary tumours, by March 1971 I had collected 53 patients, including seven children; fine needle aspiration had been used to make a diagnosis in 50. The cytological accuracy for the diagnosis of neoplasm was 100%.

The Karolinska workers (Eneroth and Zajecek, 1966) indicated how needle aspiration of salivary lesions is useful to answer:—

Is the lesion a neoplasm?
Is surgery and/or radiotherapy indicated?
How radical should surgery be?
Is pre-operative radiotherapy advisable?

**Abdominal masses**

In a lesser group of abdominal and miscellaneous masses (table 1) fine needle aspiration was precise in each case. The only unconfirmed lesion was the possible hamartoma of liver. The accompanying table refers:

Table 1

*Abdominal Masses*

| | |
|---|---|
| *Hepatomegaly* | 35 patients (25 aspirations) |
| Secondary carcinoma | 17 patients |
| Lympho-reticular disease | 2 patients |
| Hepatoma or Hamartoma | 1 patient |
| *Splenomegaly* | 6 patients |
| Reticulosarcoma | 1 patient |
| Follicular lymphoma | 1 patient |
| Lymphosarcoma | 1 patient |
| *Pancreatic Disease* | 12 patients |
| Carcinoma | 4 patients |
| Chronic pancreatitis | 3 patients |
| Normal pancreas | 5 patients |
| *Miscellaneous Aspirations* | |
| Neuroblastoma | |
| Renal carcinoma | 3 patients |
| Infarcted spleen | |

The Karlolinska workers (Van Schreeb et al, 1967), diagnose and type Grawitz tumours by renal arteriography and precise fine needle aspiration.

**Mammary lumps**

From 1967 until March 1971, 440 patients with mammary disease had been examined; 412 aspirations

Plate XLIII Breast cytology, fibroadenoma; a sheet of benign epithelium. x 400.

Plate XLIV Breast cytology, fibroadenoma; showing the distinction between epithelial (E) and sentinel cells (S). x 400.

Plate XLV Breast cytology; low grade carcinoma showing 'nuclear cannibalism' (A). x 400.

Plate XLVI Mucoid carcinoma of the breast; the stroma (P) was P.A.S. positive. x 160.

Plate XLVII Cytology equivalent of Plate XLVI. The tumour is low grade; the mucoid stroma is clear (P). x 400.

Plate XLVIII High grade breast cancer, showing a condensed reticular chromatin. x 400.

Plate XLIX High grade breast cancer, showing a broken up reticular pattern and prominent nucleoli; 'Hodgkin type'. x 400.

Plate L Lymph node imprint; showing a range of lymphoid cells and a 'blast' cell (B) x 400.

Plate LI Multiple myeloma; aspiration smear of a lesion in the clavicle. x 400.

Plate LII Aspiration smear of scalene lymph node showing 'oat cell' carcinoma. x 400.

Plate LIII Histology of node from plate LII; typical 'oat cell' carcinoma. x 200.

were performed. The accuracy of fine needle aspiration for carcinoma was 96%; for benign lesions, 94% (Plates XLIII—XLIX). This accords fine needle aspiration an acceptable place in clinical practice (Webb, 1970).

Mammary cytology is valuable on several counts:

1. To confirm a clinically likely carcinoma, obviating the need for frozen section and biopsy.
2. To clarify inflammatory swellings and four-quadrant lesions where surgical biopsy is preferably avoided and sometimes contra-indicated.
3. To elucidate, together with mammography, vague breast lumps and 'lumpiness'.
4. To assign priority in surgical management.

Scrape smears and cytology are also the ideal means whereby to confirm Paget's disease of the nipple.

**Lympho-reticular disease**

The diagnosis of lympho-reticular disease (Plates L-LIII) presenting in lymph nodes, spleen, liver or skeleton is greatly facilitated by needle aspiration and im-

Plate LIV Scrape smear cytology, Marjolin's ulcer of leg; typical keratinised malignant squames (S). x 400.

Plate LV Aspiration smear seminoma of testis showing malignant cells, lymphocytes and one epithelioid cell (E). The background—'tiger-substance' (T) is characteristic. x 400.

print smears. Splenic aspiration may reveal Hodgkin's disease when the clinical presentation is otherwise obscure without lymphadenopathy.

**Other conditions**

Fine needle aspiration, performed transrectally, was introduced by Franzén Giertz and Zajicek (1960) to elucidate the diagnosis of prostatic cancer. The method is revolutionary and of great clinical importance. The author has not made a special study of prostatic disease but the technique is regularly employed as the occasion demands. Preparations from other conditions are illustrated in plates LIV and LV.

**Conclusion**

The advantages of fine needle aspiration with a

20 ml syringe and a needle size less than 0.8 mm may be itemised as follows:

- Convenient
- Cheap
- Expeditious
- Atraumatic
- Repeatable
- Contributory

In some fields, the accuracy is astonishingly high; even when not quite so precise it has always been found to contribute to the clinical diagnosis. The incidence of failed aspirations should, in expert hands, be less than 5%.

Edward Long Fox would, I feel sure, have wished his lecturer to issue a challenge to his audience, hoping to stimulate someone to further discovery. Two more cases might provide me with this opportunity.

**Case 3**

A middle-aged woman presented with a parotid swelling from which surgical biopsies had revealed a histological problem—eventually labelled benign lympho-epithelial lesion. Two years later the parotid swelling had returned and occluded the external auditory meatus. Fine needle biopsy showed Hodgkin cells. Radiotherapy caused the mass to vanish. A year later a contralateral cervical lymph node, histologically assessed elsewhere, confirmed Hodgkin's disease.

**Case 4**

On Christmas Eve 1962, I obtained smears from a repeat surgical biopsy from large cervical lymph nodes, in a woman thirty weeks 'great with child'. The smears showed Reed-Sternberg cells; the first time I had ever seen them.

The bizarre and fascinating changes seen in the Reed-Sternberg or Hodgkin cell (Plate LVI) are so much more clear and impressive on a cytological

Plate LVI Hodgkin's disease, aspiration smear of cervical lymph node: Reed-Sternberg cells (R). x 400.

smear. The stages between this cell and a reactive blast cell or epithelioid cell are equally riveting (Bessis, 1956). Cytology shows chromatin changes in cells smaller than normal blast cells.

What do these changes mean? Their elucidation must hold a clue to this fascinating disease process which cytologically so often appears to be a bizarre distorted reactive cellular process. Perhaps someone here can find the answer:—a Nobel Prize should await him.

Since Hooke's day we have learned much in the study of cells by microscopy, yet in so many instances we still see 'as in a riddle'—we do not understand. Or as St. Paul chides us in Corinthians—'Through a glass darkly'.

REFERENCES

ABERNETHY, J. (1817), The Surgical Works, 2nd Vol., p. 261. Longman.

BAMFORTH, J. (1966). Cytological diagnosis in medical practice. Churchill.

BEALE, L. S. (1954). The microscope and its application to clinical medicine. Highley.

BESSIS, M. (1956). Cytology of the blood and blood forming organs. Tr. Ponder. Grune and Stratton.

BENNETT, J. H. (1849). On cancerous and cancroid growths. Sutherland and Cox.

BICHAT, M. F. X. (1802). Traite des membranes. Richard.

BLAND-SUTTON, J. (1822). Tumours, Innocent and Malignant. 7th Ed. Cassel.

BLOODGOOD, J. C. (1931), Biopsy in the treatment of malignancy. Journal of Laboratory and Clinical Medicine, **16**, 692.

BOSS, J. (1965), Hooke, the microscope and 300 years of cells: 1665-1965. Open lecture, University of Bristol Medical School.

BRADBURY, S. (1967). The evolution of the mircroscope. Pergamon.

BUDD, W. (1841-2), Remarks on the pathology and causes of cancer. Lancet, **42**, 266.

CAMERON, G. R. (1952), Pathology of the Cell. Oliver and Boyd.

CARDOZO, P. L. (1971), Two outcasts in the realm of clinical cytology. Goldblatt Lecture II. 4th International Congress of Cytology, London.

CASTELAIN, G. et CASTELAIN C. (1957). Possibilités et limites du cytodiagnostic per-operatoire chirurgie des tumeurs. Press medicale. **65**, 1173.

CONN, H. J. (1948). The history of staining. Biotech. Pub.

COOMBS, C. F. (1925), Aetiology of Cardiac Disease, 14th Long Fox Lecture.

COOPER, Sir A. P. (1829), Illustrations of diseases of the breast. Part I. Longman.

DAVIES, J. N. P. (1962), Aspects of the Geographic Pathology of Cancer. 51st Long Fox Lecture.

DUDGEON, L. S. and PATRICK, C. V. (1927), A new method for the rapid microscopical diagnosis of tumours. British Journal of Surgery, **15**, 250.

DUDGEON, L. S. and BARRETT, N. R. (1934), The examination of fresh tissues by the wet film method. British Journal of Surgery, **22**, **4**.

ENEROTH, C. M., FRANZÉN, S and ZAJICEK, J. (1967), Aspiration biopsy of salivary gland tumours. A critical review of 910 biopsies. Acta cytologica **11**, 470.

ERICHSEN, J. E. (1853). The Science and Art of Surgery, 1st Ed. Walton and Maberly.

FRANZÉN, S., GIERTZ, G., and ZAJICEK, J. (1960). Cytological diagnosis of prostatic tumours by transrectal aspiration biopsy. British Journal of Urology, **32**, 193.
GODWIN, J. T. (1955-56), Aspiration biopsy, technique and application. Annals of New York Academy of Sciences, **63**, 1348.
GRIEG, E. D. W. and GRAY, A. C. H. (1904), Note on the lymphatic glands in sleeping sickness. British Medical Journal **1**, 1252.
GUTHRIE, C. G. (1921), Gland puncture as a diagnostic measure. Bulletin of Johns Hopkins Hospital, **32**, 266.
Von HAAM, E. (1962), A comparative study of the accuracy of cancer cell detection by cytological methods. Acta cytologica **6**, 508.
HARRISON, A. J. (1906), Dermatitis from without and dermatitis from within, 3rd Long Fox Lecture.
HODGKIN, Dr. and LISTER, J. J. (1827), Notice on some microscopical observations of the blood on animal tissues. Philosophical Magazine, **2**, 130.
HOME, Sir E. (1830), A short note on the formation of tumours, Longman.
HOOKE, R. (1745), Micrographia restaurata or the copper plates of Dr. Hooke's wonderful discoveries. Bowles.
HOOKE, R. (1665), Micrographia.
HUGHES, A. (1959), A History of Cytology. Abelard-Schuman.
KISCH, B. (1954). Forgotten leaders in modern medicine. Transactions of American Philosophical Society **44**, 139.
KÜN, M. (1847), A new instrument for the diagnosis of tumours. Monthly Journal of Medical Science, **7**, 853.
McGRAW, A. B. and HARTMANN, F. W. (1933). Present status of the biopsy. Journal of American Medical Association, **101**, 1205.
MORGAGNI, G. B. (1822), Seats and causes of diseases. Tr. Cooke, W. 2 vols. London.
MÜLLER, J. (1836). Ueber den feineren bau und die Formen der Krankhaften Geschwultzte. Riemer.
NICHOLSON, G. W. (1922), Studies on tumour formation, IV. Guy's Hospital Report, **72**, 352.
NIXON, J. A. (1930), Influence of food on disease, 19th Long Fox Lecture.
PAGET, J. (1853), Lectures on tumours, Longman.
PAGET, J. (1853), Lectures on surgical pathology, vol. 2. Longman.
PRICE, C. H. G. (1973), A critique of Ewing's tumour of bone, p. 177. In 'Bone—certain aspects of neoplasia', Colston Symposium Papers No. 24, Ed. C. H. G. Price and F. G. M. Ross.
PRICHARD, A. (1863), Ten years of operative surgery in the provinces, 1850-1860, 875 operations. Part 2. Richards.
SCHLEIDEN, M. J. (1847), Contributions to phytogenesis. Tr. Smith H. Sydenham Society.
Van SCHREEB, T., FRANZEN, S., and LJUNQUIST, A. (1967), Renal adenocarcinoma, evaluation of malignancy on a cytological basis. Scandinavian Journal of Urology and Nephrology **1**, 265.
SCHWANN, T. (1847), Microscopical researches into the accordance in the structure and growth of animals and plants. Tr. Smith, H. Sydenham Society.
SKEY, F. C. (1850), Operative Surgery. Churchill.
SÖDERSTRÖM, N. (1966), Fine needle aspiration biopsy. Almqvist and Wiksell.
SMITH, G. MUNRO (1917), History of the Bristol Royal Infirmary, Arrowsmith.
Van der STAR, P. (1953), Descriptive catalogue of the simple microscopes in the Riksmuseum voor de Gescheidenis der Natuurwetens Schappen. Leiden.
STEDMAN'S MEDICAL DICTIONARY (1972). 22nd Ed. Williams and Wilkins.
VIRCHOW, R. (1860), Cellular Pathology. Tr. Chance, F. 2nd Ed. Churchill.
WARTHIN, A. S. (1929) Papillary Cystadenoma Lymphomatosum. Journal of Cancer Research, **13**, 116.
WEBB, A. J. (1970), The diagnostic cytology of breast carcinoma. British Journal of Surgery, **57**, 260.
WEBB, A. J. (1973), The cytological diagnosis of round-cell tumours in 'Bone, certain aspects of neoplasia'. Colston Symposium Papers No. 24. p. 251. Ed. Price, C. H. G. and Ross, F. G. M. Butterworths.
WRIGHT, D. H. (1963), Cytology and histochemistry of the Burkitt Lymphoma. British Journal of Cancer, **17**, 50.

## Figures and Tables

**Plate XXXVII f1:**
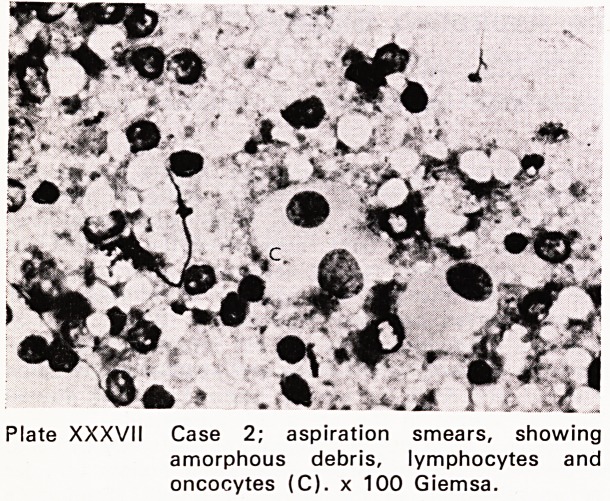


**Plate XXVIII f2:**
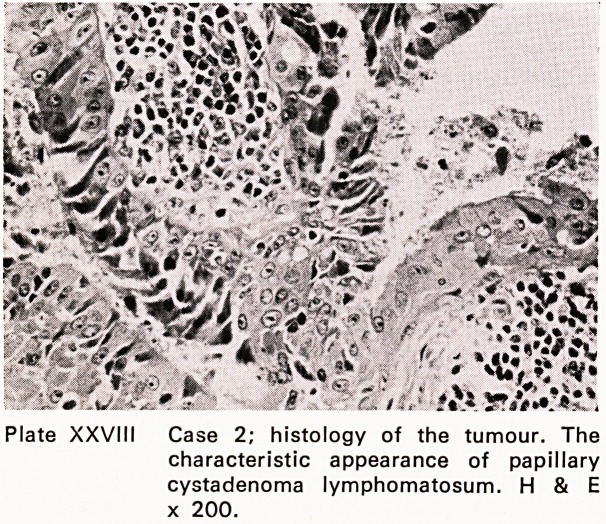


**Plate XXXIX f3:**
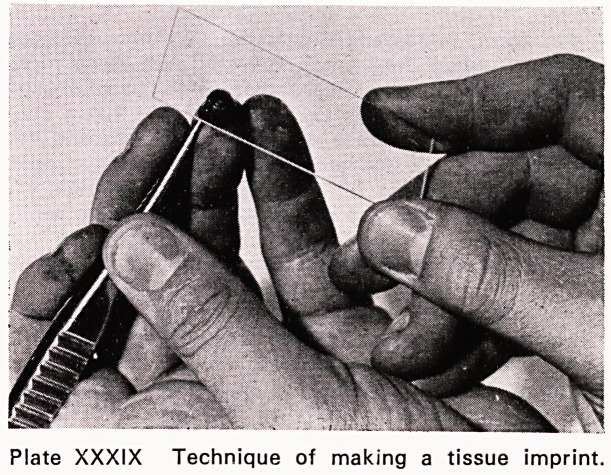


**Plate XL f4:**
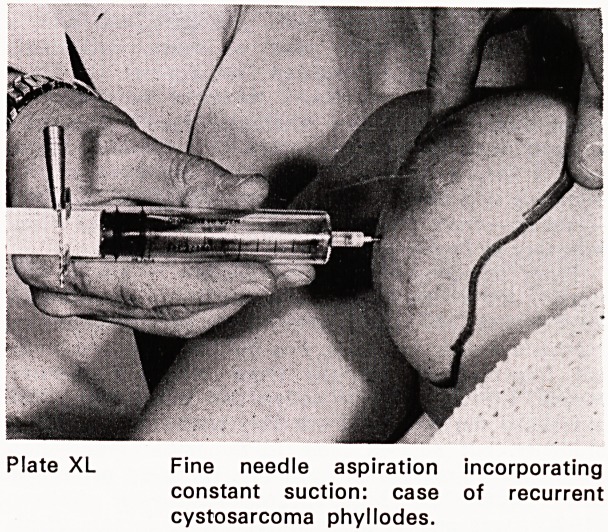


**Plate XLI f5:**
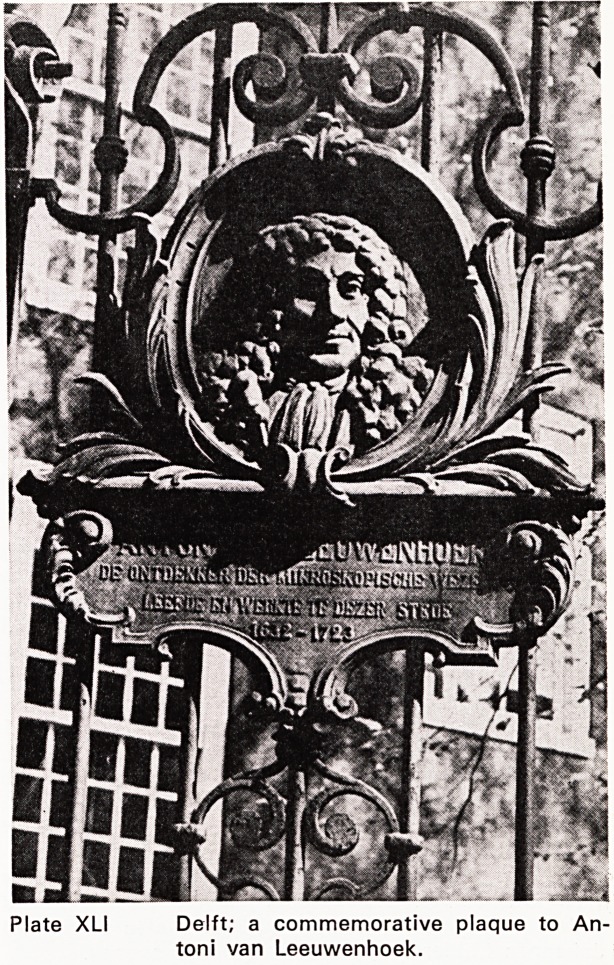


**Plate XLII f6:**
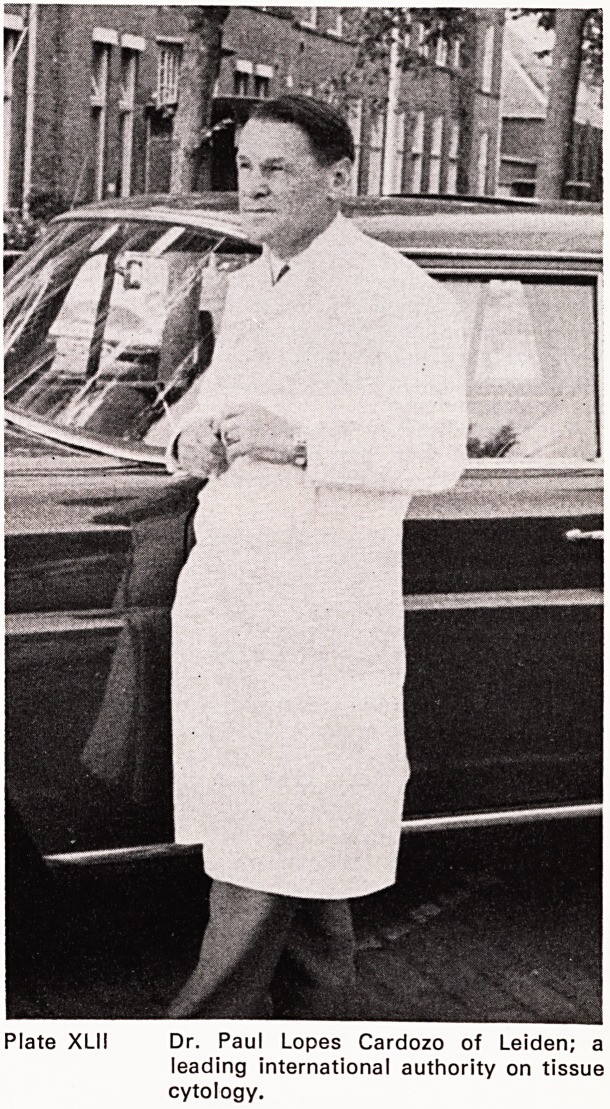


**Plate XLIII f7:**
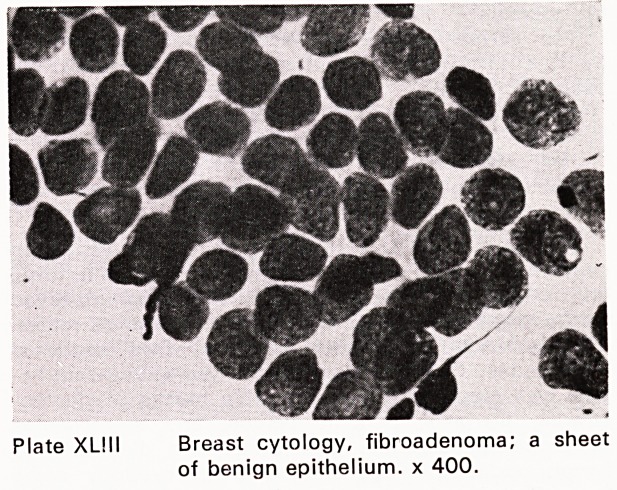


**Plate XLIV f8:**
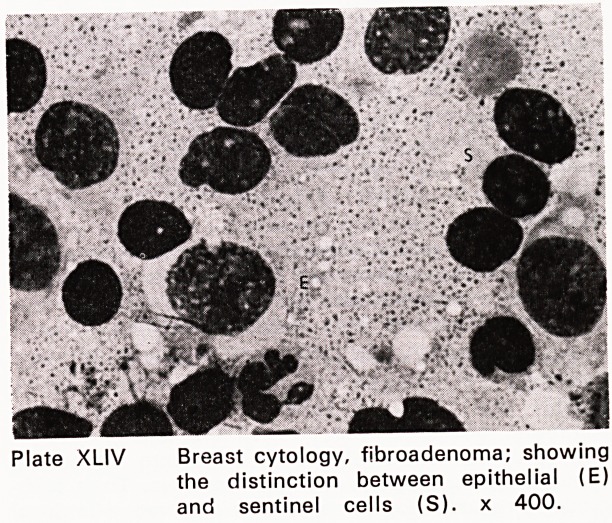


**Plate XLV f9:**
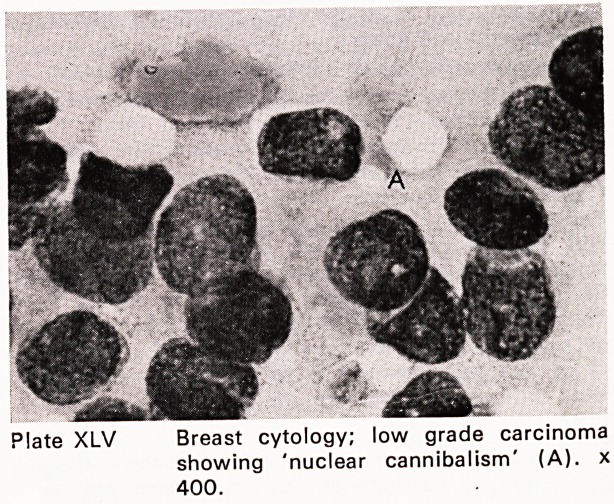


**Plate XLVI f10:**
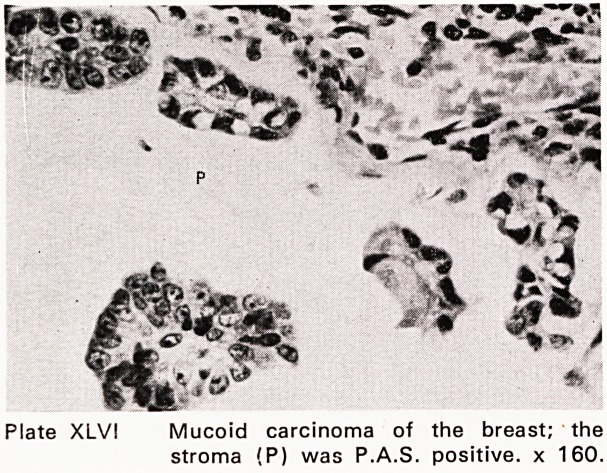


**Plate XLVII f11:**
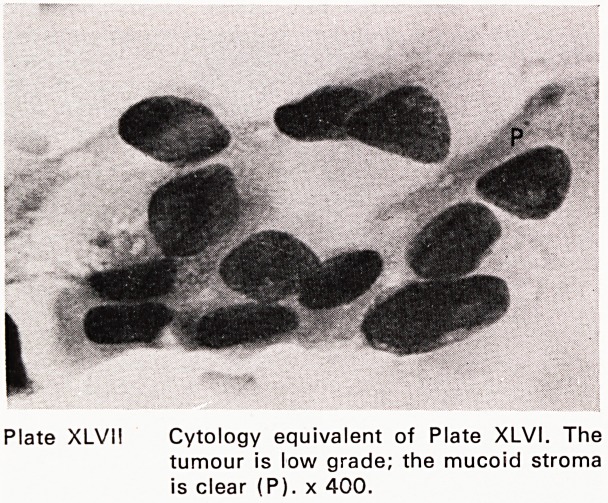


**Plate XLVIII f12:**
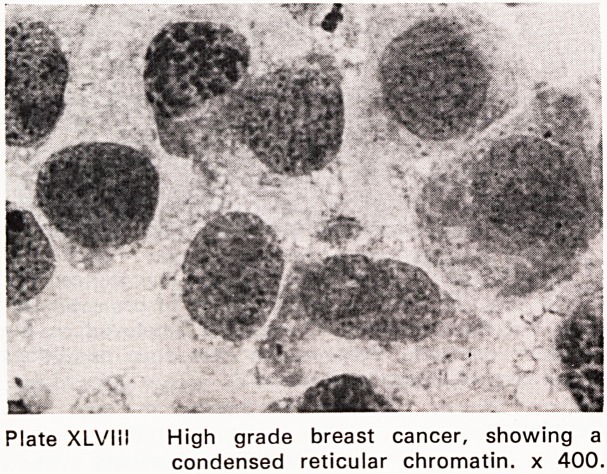


**Plate XLIX f13:**
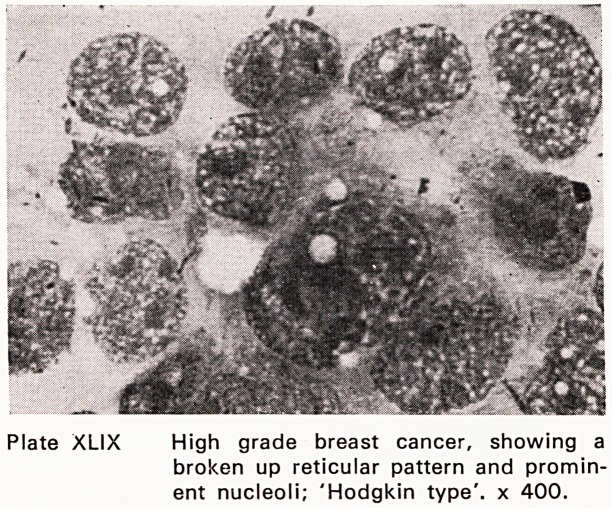


**Plate L f14:**
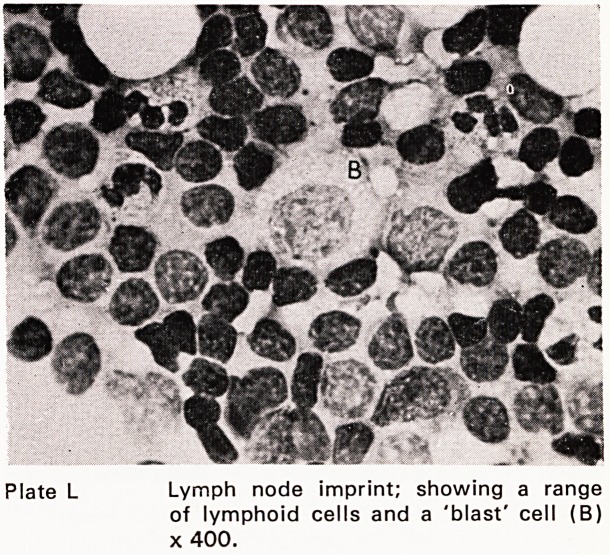


**Plate LI f15:**
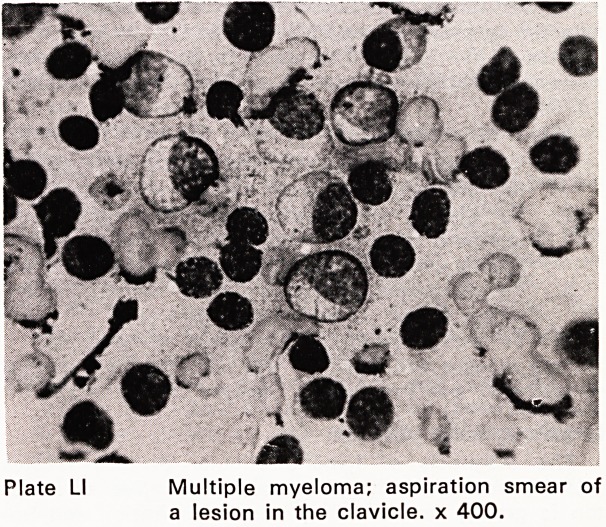


**Plate LII f16:**
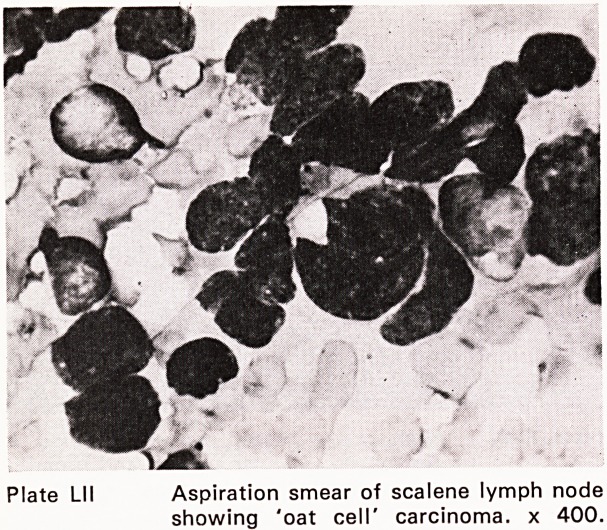


**Plate LIII f17:**
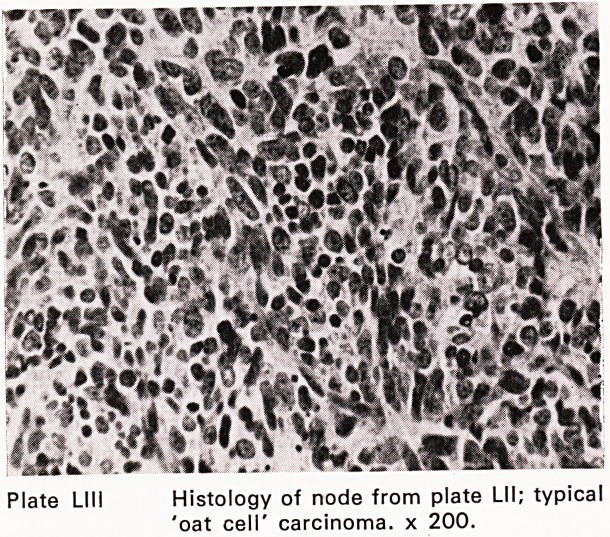


**Plate LIV f18:**
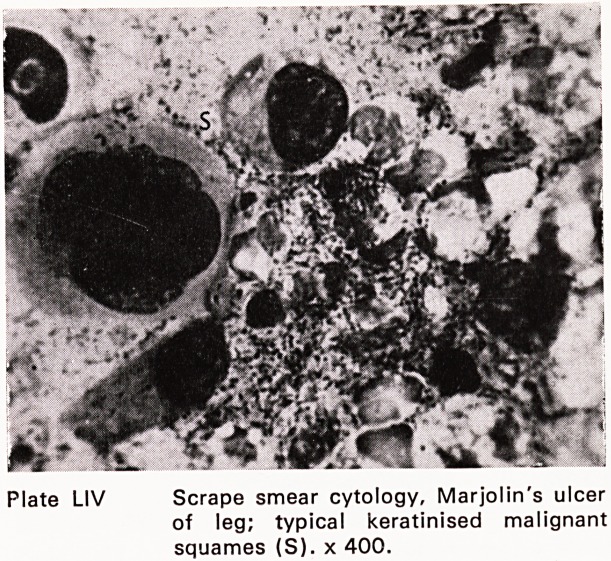


**Plate LV f19:**
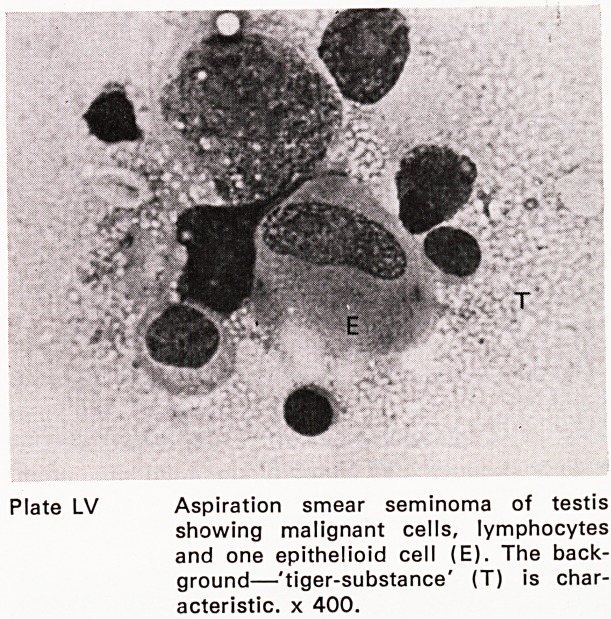


**Plate LVI f20:**